# Use of unmanned ground vehicle systems in urbanized zones: A study of vector Mosquito surveillance in Kaohsiung

**DOI:** 10.1371/journal.pntd.0011346

**Published:** 2023-06-08

**Authors:** Yu-Xuan Chen, Chao-Ying Pan, Bo-Yu Chen, Shu-Wen Jeng, Chun-Hong Chen, Joh-Jong Huang, Chaur-Dong Chen, Wei-Liang Liu

**Affiliations:** 1 National Mosquito-Borne Diseases Control Research Center, National Health Research Institutes, Miaoli, Taiwan; 2 Department of Biotechnology and Bioindustry Sciences, National Cheng Kung University, Tainan, Taiwan; 3 Department of Health, Kaohsiung City Government, Kaohsiung, Taiwan; 4 Graduate Institute of Science Education & Environmental Education, National Kaohsiung Normal University, Kaohsiung, Taiwan; 5 National Institute of Infectious Diseases and Vaccinology, National Health Research Institutes, Miaoli, Taiwan; 6 Institute of Molecular Medicine, College of Medicine, National Taiwan University, Taipei, Taiwan; 7 Department of Medical Humanity and Education, College of Medicine, Kaohsiung Medical University, Kaohsiung, Taiwan; 8 Sanmin District Public Health Center, Department of Health, Kaohsiung City Government, Kaohsiung, Taiwan; Faculty of Science, Mahidol University, THAILAND

## Abstract

Dengue fever is a vector-borne disease that has become a serious global public health problem over the past decade. An essential aspect of controlling and preventing mosquito-borne diseases is reduction of mosquito density. Through the process of urbanization, sewers (ditches) have become easy breeding sources of vector mosquitoes. In this study, we, for the first time, used unmanned ground vehicle systems (UGVs) to enter ditches in urban areas to observe vector mosquito ecology. We found traces of vector mosquitoes in ~20.7% of inspected ditches, suggesting that these constitute viable breeding sources of vector mosquitoes in urban areas. We also analyzed the average gravitrap catch of five administrative districts in Kaohsiung city from May to August 2018. The gravitrap indices of Nanzi and Fengshan districts were above the expected average (3.26), indicating that the vector mosquitoes density in these areas is high. Using the UGVs to detect positive ditches within the five districts followed by insecticide application generally yielded good control results. Further improving the high-resolution digital camera and spraying system of the UGVs may be able to effectively and instantly monitor vector mosquitoes and implement spraying controls. This approach may be suitable to solve the complex and difficult task of detecting mosquito breeding sources in urban ditches.

## Author summary

In recent years, many countries have used new technologies to prevent dengue fever, among which unmanned vehicle systems are the most promising. We combined a crawling robot, wire-controlled cable car and real-time monitoring system into an unmanned ground vehicle system (UGV) to conduct ecological surveys of vector mosquitoes in sewers (ditches). The study found that the ditches in Kaohsiung city were a breeding source for dengue vector mosquitoes. Inspection by UGVs determined that 12 of the 58 examined ditches contained *Aedes* mosquitoes in stages from larvae to adult. Positive ditches were then sprayed with insecticides or flushed with high-temperature water jets to prevent the proliferation of mosquitoes. The widespread use of UGVs can potentially eliminate some of the breeding sources of vector mosquitoes, thereby reducing the annual prevalence of dengue fever in Kaohsiung city.

## Introduction

Dengue fever is an infectious disease caused by the dengue virus and is one of the world’s most rapidly spreading mosquito-borne viral diseases. The World Health Organization estimates that 390 million people worldwide are infected with this virus annually [1,2]. Dengue fever-prone areas are concentrated in tropical and subtropical countries, and several mosquito species in the genus *Aedes* are the vector that carries and transmits the viruses to humans by biting [3,4]. *Aedes* mosquitoes are not only the main vector of dengue, but also of other arboviruses of growing importance, such as chikungunya, yellow fever and zika. Taiwan is located in a subtropical region with high temperatures and high humidity, which causes seasonal dengue fever in Southern Taiwan almost every summer [5,6]. Since there is currently no effective vaccine against dengue, the control and elimination of *Aedes* mosquitoes constitutes a perennial problem calling for implementation of effective entomological surveillance combined with the development of new vector control measures.

Surveillance is a key component of any dengue prevention and control program. Unmanned aerial vehicles (UAVs) have recently been used in mosquito control monitoring in many countries [7–10]. Although the UAV is a novel technology, it still lacks the ability to monitor and analyze urban sewers. The use of unmanned ground vehicle systems (UGVs) [11], also known as unmanned remote-controlled crawler vehicles, coupled with a high-quality camera system to monitor the density of vector mosquitoes in sewers (ditches), may constitute a suitable technological approach to epidemic prevention. In addition to locating mosquito larval habitats, UGVs can also provide real-time images to improve the detection and identification of mosquito larvae and adults, and carry out the eradication and prevention of vector mosquitoes. Widespread use of advanced UGV designs may be effective in protecting the population from mosquito-borne diseases.

Reports by the World Health Organization and other researchers indicate that dengue fever tends to spread easily in urban and semi-urban areas [12–14]. Kaohsiung in Southern Taiwan is a highly urbanized city. The large-scale landscape changes and population agglomeration accompanying the urbanization process not only play an important role in the long-term changes in biodiversity, but also generate a considerable number of potential breeding sources of vector organisms [15,16]. Residential migration and urban-rural construction have caused environmental and ecological changes, which were found to cause substantial variations in the ecology of vector mosquito breeding sources. In the past, it was thought that ditches did not act as mosquito breeding grounds, but traces of *Aedes* mosquitoes are now reported from such locations [17,18]. A few decades ago, the ditches in Kaohsiung city would not have constituted an important factor in the dengue epidemic, but in recent years they have become a driver for outbreaks of dengue fever. In fact, vector mosquito monitoring by the municipal government repeatedly found that ditches contributed up to 18.7% of positive containers for dengue vector mosquito breeding, ranking second-highest among hidden breeding sources [19] (S1 Fig). Even though the sanitary sewage and sewer systems in urbanized Kaohsiung have been significantly improved, and the service rate now exceeds 80%. Domestic sewage and oily wastewater flows into dedicated sewage systems, so the ditches that used to discharge household wastewater near domestic units are no longer polluted. But these ditches with clean water have gradually become potential habitats for Aedes larvae. Therefore, the interaction between larval habitats and urbanization processes is another important topic for exploring dengue transmission.

Effective targeting of mosquito breeding sites in urban disease control programs requires up-to-date information on their location. A multi-purpose UGV equipped with various sensory equipment can quickly analyze and visualize these sites in urban sewer/ditch environments. Although UGV sensors have improved in detecting vector habitats, field verification is still necessary. In this study, a simple UGV was used to inspect ditches in five administrative areas of Kaohsiung city and evidence of vector mosquitoes was found. This further verified that the ditches were indeed one of the breeding sources of vector mosquitoes in Kaohsiung after urbanization. In future, UGVs equipped with high-resolution digital camera and insecticide spraying systems will be utilized to detect and eliminate vector mosquitoes, potentially improving the work of front-line epidemic prevention teams.

## Material and methods

### Description of study area

**Fig 1:**
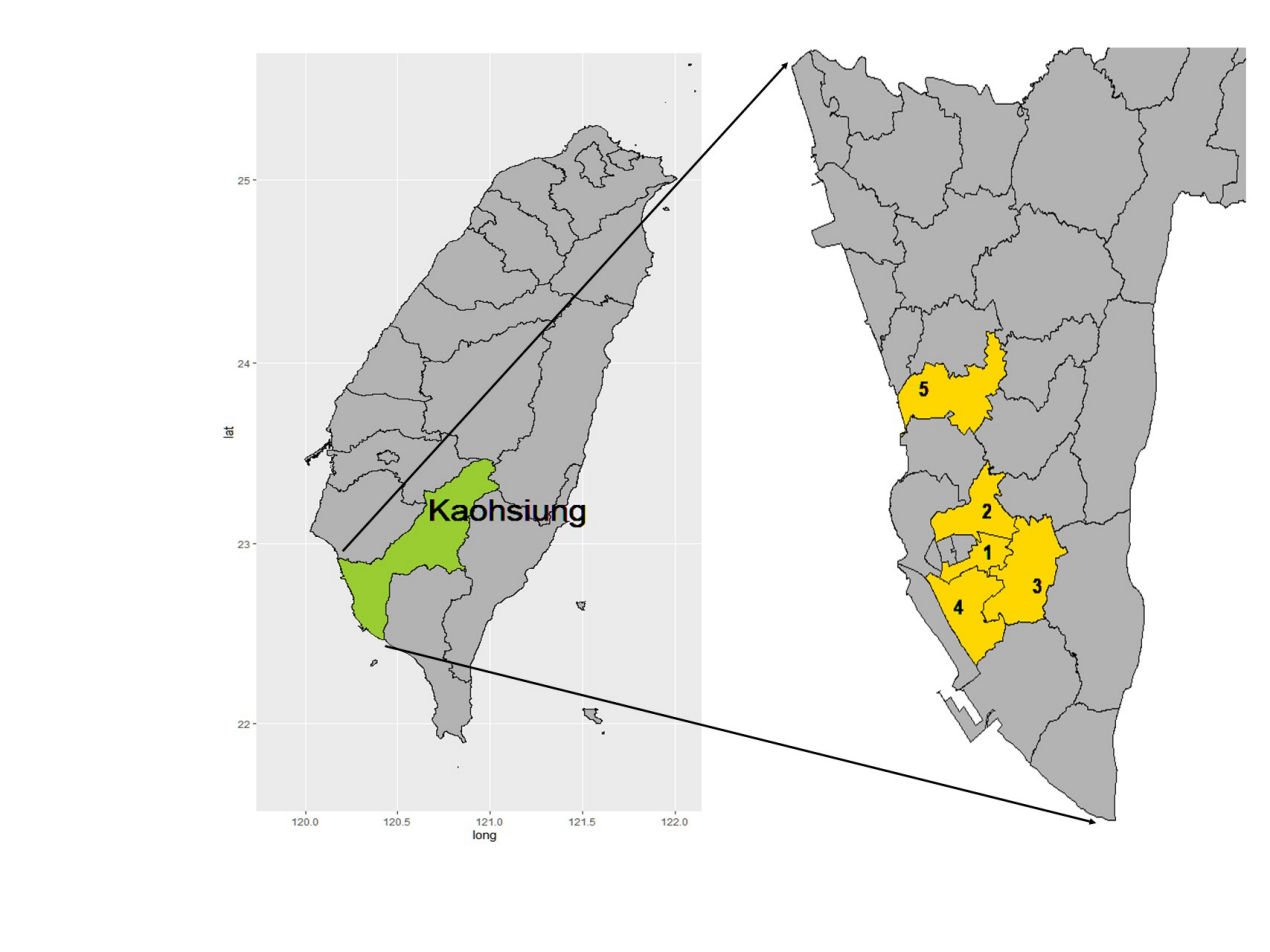
Surveillance areas used in this study. **Kaohsiung city is shown in the left panel.** The five urban districts included in this study are shown in the right panel. 1: Lingya district, 2: Sanmin district, 3: Fengshan district, 4: Qianzhen district, 5: Nanzi district. The full map of Taiwan and a detailed map of the Kaohsiung administrative district were obtained from https://maps.nlsc.gov.tw/. The maps were created using the ’ggplot2’ package in R".

Kaohsiung city is one of the largest cities in Southern Taiwan (Fig 1), with a population of 2.7 million. The average temperature is 26°C with ∼1,800 mm of rainfall per year, providing an ideal environment for *Aedes* mosquito reproduction. During May to August 2018, field monitoring experiments were conducted in five districts (Lingya, Sanmin, Fengshan, Qianzhen, and Nanzi) that have a history of dengue fever outbreaks over the past 10 years. The Kaohsiung city epidemic prevention unit noted that these areas have a high proportion of mosquito breeding sources in ditches, and gravitraps were placed to focus on these ditches (S1 Fig). Positive ditches were defined as those containing vector mosquito larvae or adults. The study area is a community of mixed old and new buildings containing residential, commercial and park areas.

### UGV machine and inspection process

**Fig 2:**
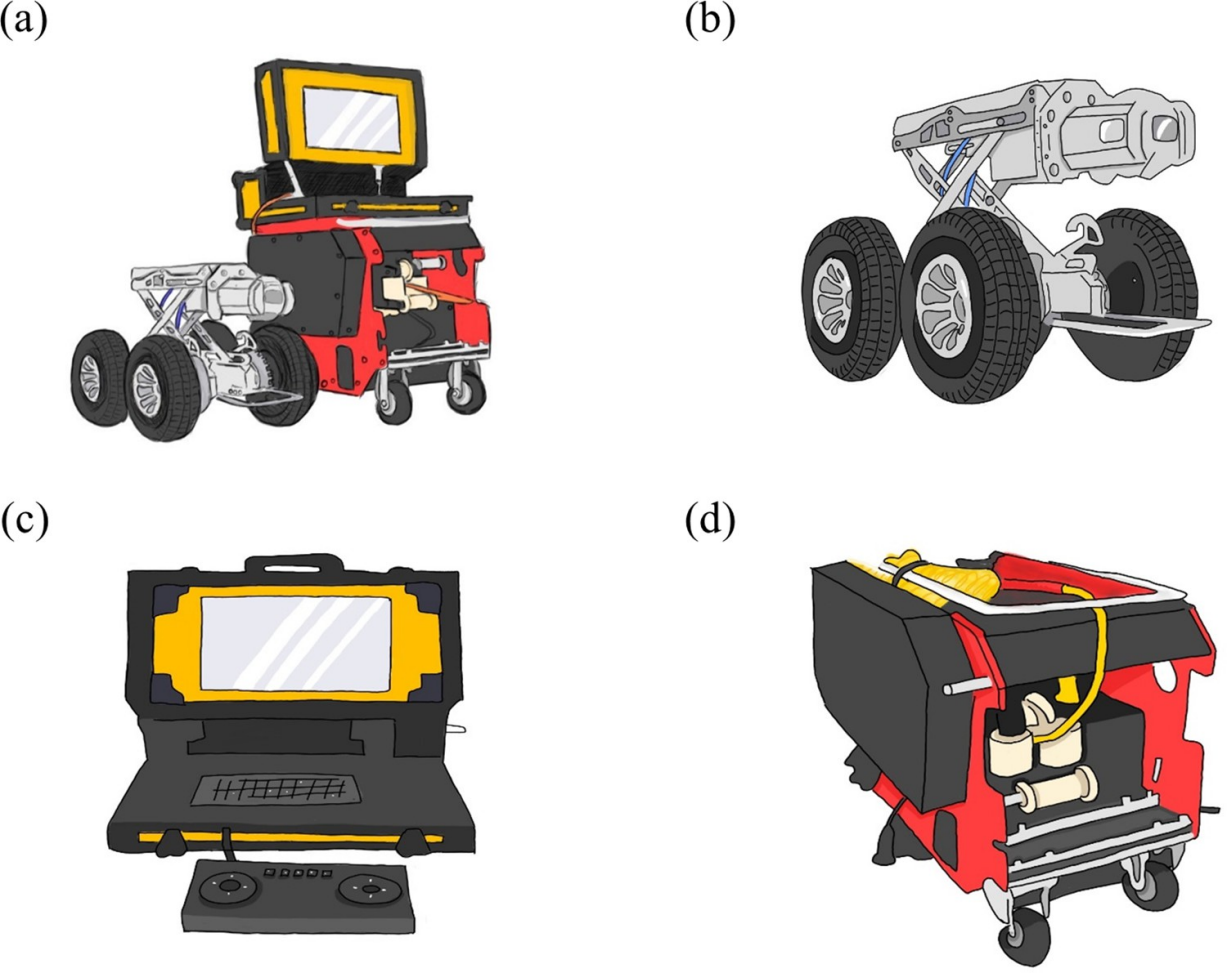
Schematic diagrams of UGV equipment. (a) The UGV consists of three main units, including (b) crawling robot, (c) real-time monitoring system and (d) wire-controlled cable car. The basic equipment accessories are shown in supplementary S1 Table.

The UGV equipment used for ditch inspection operations consisted of three main units: the crawling robot, wire-controlled cable car and real-time monitoring system. The basic equipment accessories are shown in Fig 2 and S1 Table. In addition to the arrangement of LED lighting equipment at the front of the machine, it was also equipped with a high-resolution digital camera system (> 250,000 pixels) for real-time monitoring. It could rotate 360° axially and 270° horizontally. The car body carried an electric lifting platform, a rear-mounted reverse imaging system and a rear-view light source, and had an anti-overturning design.

The investigation covered five administrative regions in Kaohsiung City suspected to be hotspots for vector mosquito ditches. Each ditch was inspected over a range of 50 to 100 meters. The ditches were all located on both sides of the lane, with a width less than 4 meters, and the surrounding buildings were less than 10 stories high, comprising a mixture of old bungalows and new tall buildings. As the health department considers this area to be a potential starting point for dengue outbreaks, the density of vector mosquitoes must be closely monitored. The ditches are covered with ventilation holes every 10 meters or so to allow entry and exit of UGVs. The investigation was conducted using digital cameras or video recording without interrupting the video recording process. The crawling robot maintained a steady speed of not more than 5 m/min. The UGVs inspection time for each entry and exit of the ditch was set for about 10 to 20 minutes, with a daily inspection time not exceeding 5 hours. The investigation records included on-site photos, recorded location of stagnant water, and the presence of larvae, as determined at the scene or during follow-up video review. The survey video recorded the entire investigated stretch, including the starting and ending points and obvious landmarks outside the ditch manhole cover, as well as GPS coordinates.

### Trap positioning

Gravitrap is a simple trap designed to lure the female mosquitoes for oviposition by providing water at the bottom of the trap. The upper body was composed of a simple black cylindrical trap with adhesive material on the inner surface to capture ovipositing female *Aedes* mosquitoes [20]. The gravitraps in the study area were placed on the ground, mainly concentrated in the shaded areas near the houses close to the ditch, at a distance of about 5 meters. However, a few traps were also placed 50 meters away. The adult mosquito populations were continuously monitored with these traps from May to August 2018, with data collected over 7-day trapping periods. The captured mosquitoes from each trap were collected, frozen, and transported to specialists for microscopic examination of morphology for species and sex identification.

### Positive ditch prevention control methods

**Fig 3:**
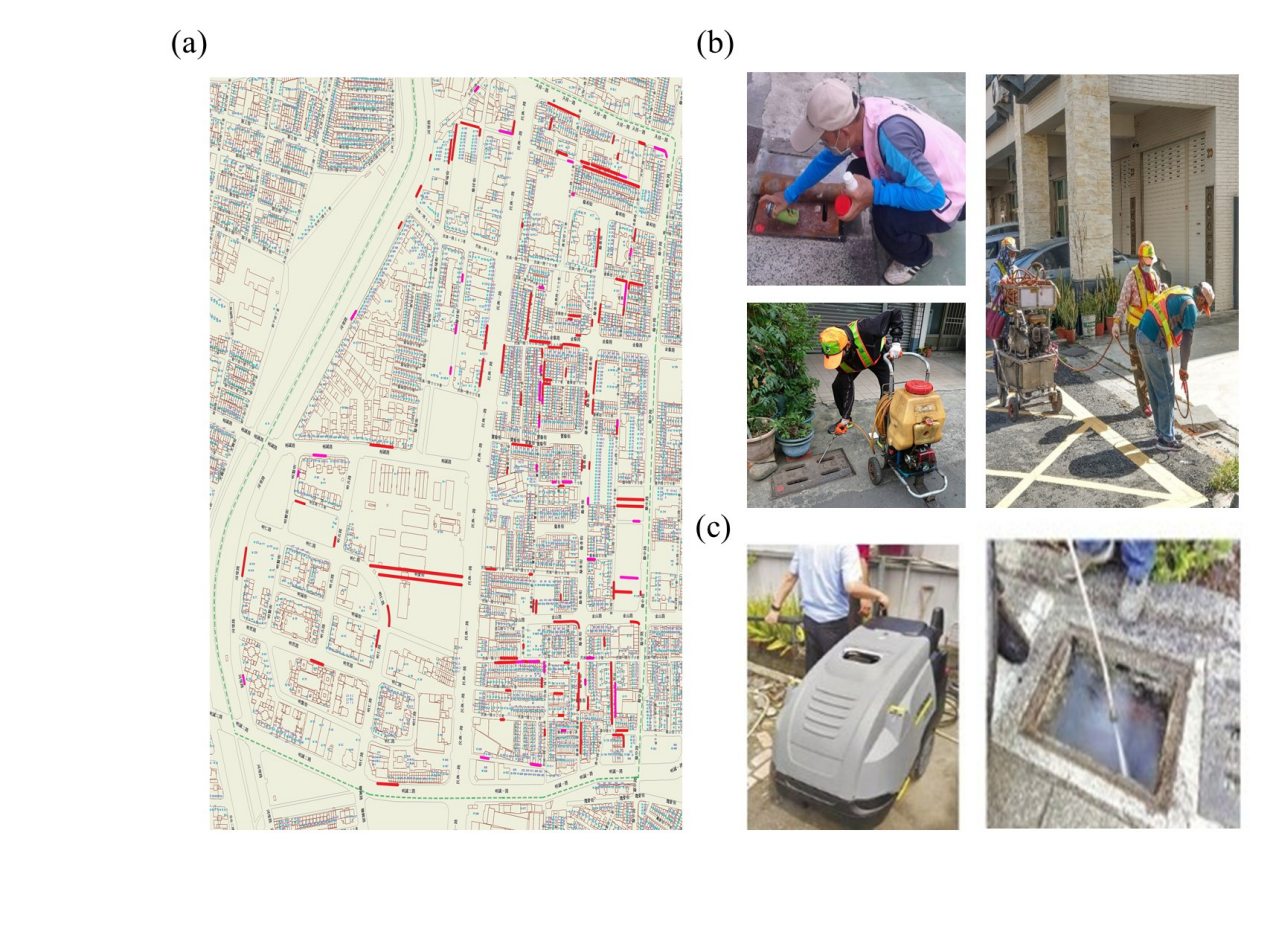
Prevention and control actions of Kaohsiung city’s epidemic prevention personnel after UGV inspection of ditches. (a) Positive ditch marked (red line) in Dingtai-Li of the Sanmin district after UGV inspection (map from: https://maps.nlsc.gov.tw/). (b) Marking and spraying insecticides and (c) using strong hot water jet to flush the walls of positive ditches to eliminate mosquito larvae and eggs.

For demarcated positive ditches, further removal of breeding sources and spraying were implemented to reduce the density of vector mosquitoes (Fig 3). The epidemic prevention team will used insecticides, such as Permethrin, Deltamethrin, Cypermethrin and other chemical drugs to kill the breeding of vector mosquitoes. In order to avoid the long-term spraying of insecticide, which may result in drug resistance, the epidemic prevention team in some ditches case used a physical control method instead, using hot water jets to flush the ditches to kill mosquitoes. Hot water at a temperature of 70–80°C and a pressure of 3500 psi was used to flush the walls of the positive ditch and eliminate mosquito larvae and eggs.

### Trap index calculation

The gravitrap index (GI) were calculated as ratios of total female number of *Aedes* mosquitoes captured over 4 months to the number of functional traps. A weekly trap index was calculated from mosquitoes captured over a week. A functional gravitrap is defined as a fully-assembled gravitrap where the inner lining remained sticky to capture mosquitoes and water was present. Traps that were empty, missing, overturned, or had lining removed were considered non-functional and excluded [21,22].


$$Gravitrap\;index\;(GI)=\;\frac{Total\;number\;of\;female\;mosquitoes\;(Ae.aegypti+Ae.albopictus)\;caught\;}{Total\;number\;of\;functional\;gravitrap}$$

### Statistical analysis

The Mann-Whitney-Wilcoxon Test was used for statistical testing with a value of *p* < 0.05 considered as statistically significant for comparisons.

## Results

### Mosquito surveillance with gravitraps

Gravitraps were placed in the study area for mosquito surveillance with a minimal distance of 30 m between traps to analysis catch efficiencies of *Ae*. *aegypti* and *Ae*. *albopictus*. Table 1 shows that gravitraps captured numbers of both species of mosquitoes. During peak time points from May to August 2018, we found that gravitraps cumulatively captured greater numbers of *Ae*. *aegypti* and *Ae*. *albopictus* mosquitoes (242 vs. 189). We also observed that the traps were able to capture 1.3 times more *Ae*. *aegypti* than *Ae*. *albopictus* and more female mosquitoes were captured than males. Over the study period, total of 427 female *Aedes* mosquitoes were captured by 131 gravitraps in the five administrative districts, with an average GI value of 3.26. Fengshan and Nanzi districts had higher GIs than the average (4.21 and 3.90 respectively), while the GIs of the other three districts (Sanmin, Qianzhen, and Lingya) were lower (2.89, 3.11 and 2.53 respectively). Overall, these data demonstrate gravitrap efficacy in capturing *Aedes* mosquitoes and suggest that *Ae*. *aegypti* are more prevalent in the area than *Ae*. *albopictus*.

**Table 1:** Mosquitoes captured by gravitraps (May–August 2018). The trap index were calculated as ratios of total female number of *Aedes* mosquitoes captured over 4 months to the total number of functional gravitraps.

District	No. traps deployed	*Ae*. *albopictus*		*Ae*. *aegypti*		Total	Total females	Trap index (female)
		M	F	M	F			
**Nanzi**	**21**	**1**	**38**	**0**	**44**	**83**	**82**	**3.90**
**Sanmin**	**28**	**0**	**33**	**0**	**48**	**81**	**81**	**2.89**
**Fengshan**	**24**	**0**	**48**	**0**	**53**	**101**	**101**	**4.21**
**Qianzhen**	**28**	**1**	**40**	**1**	**47**	**89**	**87**	**3.11**
**Lingya**	**30**	**1**	**27**	**0**	**49**	**77**	**76**	**2.53**
**Total**	**131**	**3**	**186**	**1**	**241**	**431**	**427**	**3.26**

M: male; F: female

### Gravitrap show difference in capture efficiency after UGV inspection ditch

**Fig 4:**
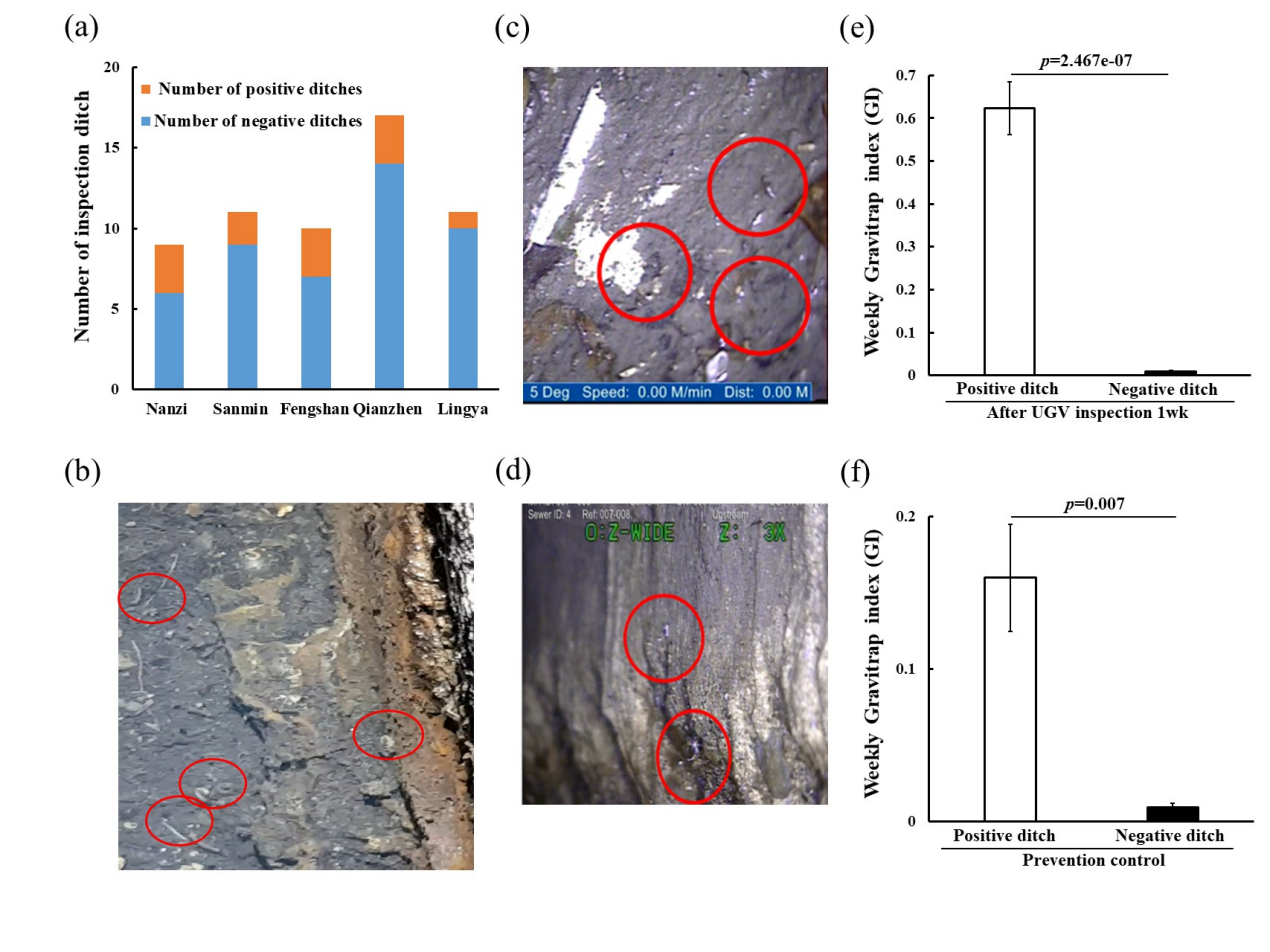
A photo taken by UGV and difference efficiency of GI in a ditch. (a) Bar chart of inspections of ditches from May to August 2018 for the five administrative districts. The images of (b) larvae, (c) pupae, and (d) adult mosquitoes, which can also be seen in the video files (S1 and S2 Video). (e) One week after inspecting the ditch of Fig 4A, analyze the GI value of the gravitrap placed around the ditch. (f) GI value of immediate prevention control methods in ditches after UGV inspection for one week. A positive ditch was defined as a ditch containing *Aedes* (larvae/pupa/adult), whereas a ditch without containing *Aedes* was a negative ditch. Prevention control: ditch has been treated with insecticides and other epidemic prevention control methods. A weekly trap index was calculated from mosquitoes captured over a week.

Previous studies have pointed out that ditches in Kaohsiung city have become an important hidden breeding source. Therefore, we used UGV to enter the ditch to observe vector mosquito activity. We inspected a total of 58 ditches within 5 administrative regions (Fig 4A). In 12 of these ditches, traces of mosquito larvae, pupae, and adult mosquitoes in flight or resting on the ditch walls were found (Fig 4B–4D and S1 and S2 Videos). These 12 ditches were marked as positive ditches, defined as ditches containing *Aedes* mosquito larvae. Positive ditches accounted for ~20.7% of all ditches inspected, which was similar to the proportion of positive containers that the Kaohsiung city epidemic prevention team had previously investigated as breeding sources of dengue fever vectors [19]. For the inspected ditches, we also placed gravitraps to monitor the density of mosquito vectors. The study found that the GI of the positive ditch was higher than the negative ditch one week after UGV inspection (0.62 vs. 0.01; Fig 4E). This inspection result is consistent with the observation in Fig 4A, UGV is suitable for inspection and the positive ditches are likely to form a breeding source for vector mosquitoes. Immediately after additional prevention control measures were taken, the GI of the positive ditch was found to decrease significantly from 0.62 to 0.19 (Fig 4E and 4F). To sum up, UGV can play an important tool in the process of vector mosquito inspection in ditches, because it can clearly find the breeding source and implement the necessary prevention control measures to reduce or kill vector mosquitoes.

### Differences in gravitrap index (GI) before and after UGV inspection

**Fig 5:**
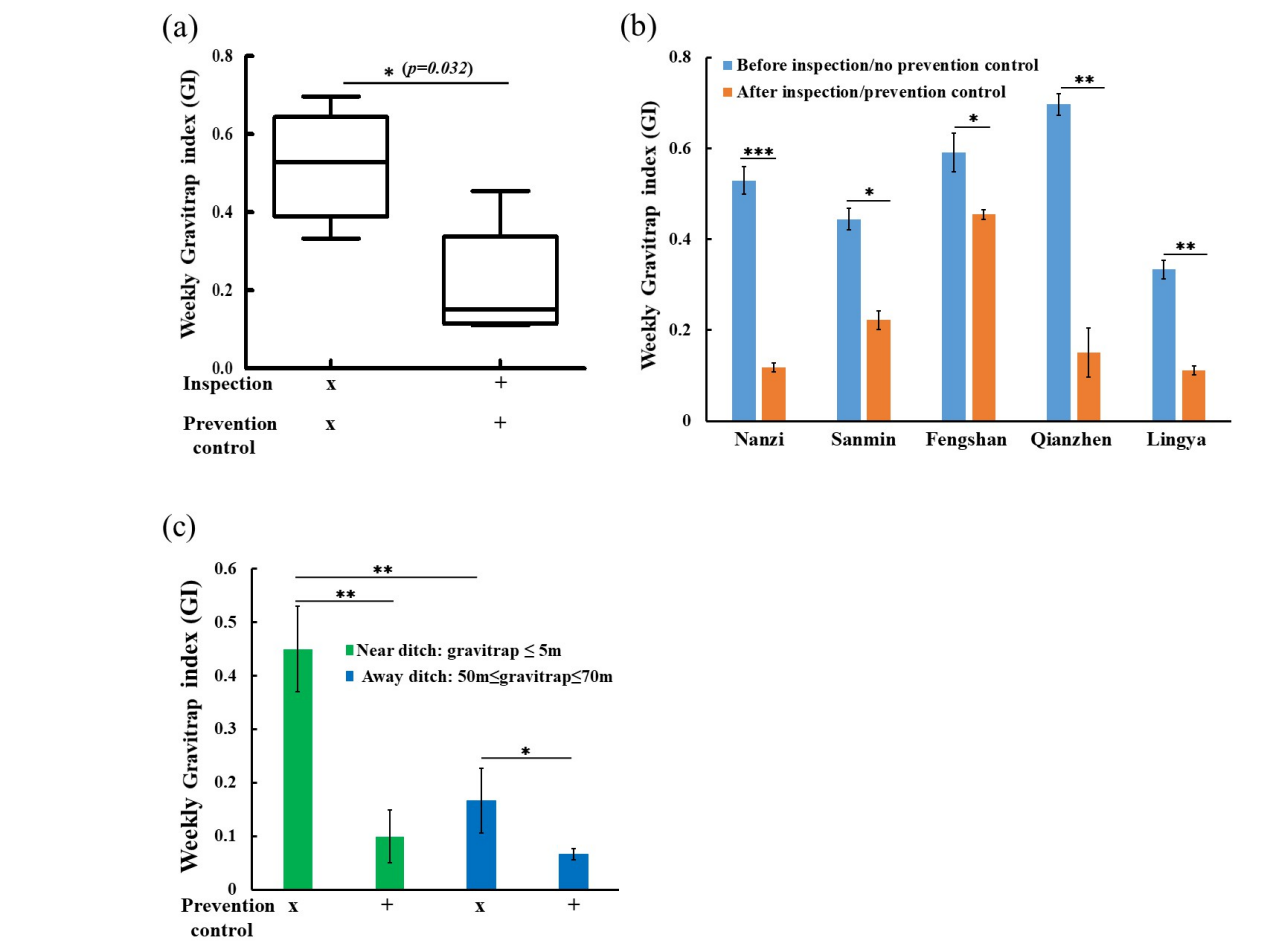
Comparison of gravitrap index (GI) values. (a) The distribution of GI values before and after UGV inspection was analyzed for all five districts and a significant difference was found (Mann-Whitney Wilcoxon test). (b) The GI values before and after inspection for each of the five districts are shown. (c) The mean values of weekly GI in the five districts are compared for areas near and far from the inspected ditch. The near-ditch trap refers to the gravitrap within 5 meters from the ditch. The far-ditch trap refers to the distance from the gravitrap greater than 50 meters and less than 70 meters, as a control group for comparison. Inspection: ditch has been inspected by UGV; Prevention control: ditch has been treated with insecticides and other epidemic prevention control methods. A weekly trap index was calculated from mosquitoes captured over a week. X: no treatment; +: treatment.

The estimates of GI were obtained by averaging data over the entire study period. We also compared GI values before and after inspection and control to determine the effectivity of the control methods. Statistical analysis showed a significant difference in GIs pooled for all five districts (0.52 vs. 0.21, *p* = 0.032, Mann-Whitney-Wilcoxon test; Fig 5A). We also aimed to examine differences in mean GI values across the five urban study areas with and without UGV inspection and preventive controls (Fig 5B). The results of the study found that the GI after inspection and prevention control in Nanzi, Sanmin, Qianzhen, and Lingya districts were significantly lower than the GI before the inspection, indicating that the prevention and control measures implemented after the inspection effectively reduced the vector mosquito density. Differences in GI between regions may be due to variations in the number of positive ditches. However, the test results in Fengshan District were disappointing. The GI did not drop significantly as expected, but reduced slowly from 0.5 to 0.45. In addition, we compared the indices of gravitraps placed close to and far from the inspection ditch after preventive controls to determine the effect of trap placement (Fig 5C). We found that the median weekly GI ranged from 0.5 to 0.72 (mean = 0.62) for traps close to the inspection ditch, and 0 to 0.03 (mean = 0.009) for traps farther from the inspection ditch. When further prevention control was included, the median weekly GI ranged from 0 to 0.29 (mean = 0.19) for traps close to the inspection ditch and 0 to 0.04 (mean = 0.009) for traps farther from the inspection ditch. These results suggest that UGVs are an important tool for urban ditches inspection, especially for the control of dengue vector mosquitoes.

## Discussion

Mosquito eggs, larvae, and pupae are all aquatic, and only the winged adults become terrestrial. Killing mosquitoes in the aquatic stages before the wings develop is therefore an efficient way to reduce the density of vector mosquitoes. Previous surveillance data indicated that ditches were one of the main vector sources for the spread of dengue fever in Kaohsiung city. In this study, UGVs were used to take photos to clearly understand the activities of *Aedes* mosquitoes in ditches, and to develop appropriate and feasible control methods that can effectively prevent a large number of vectors from breeding in these habitats in Kaohsiung city.

In recent years, the use of unmanned aerial vehicles (UAVs) in mosquito control monitoring has become more convenient and popular. Such UAVs can cover large and inaccessible areas without much difficulty, detect potential dengue breeding sites faster than humans, and capture high-resolution images and send them to online storage in real-time for efficient and fast data processing [23–26]. UGVs are capable of operating on multiple terrains and searching for targets while avoiding obstacles, making them good candidates for use in conjunction with UAVs to complement each other and search for breeding sites in different terrains. While UGVs are slower than drones and have limited communication range (line of sight, obstacles, etc.), they have higher autonomy. The UGV used in this experiment is simple and just can only be monitored (Fig 2). However, future modifications can increase its usability. If the UGV is supplemented by powerful lighting equipment and equipped with a high resolution image and video transmission system, the vector mosquito situation within the ditch can be reported in real time. In addition, control facilities (spraying systems) such as liquid insecticide equipment or ultra-low volume fog machine (ULV) equipment for killing adult mosquitoes can be installed on this crawling robot. It can also be provided with a granular spreader that contains growth inhibitors to impede vector mosquito larvae growth. When a UGV is used in conjunction with a UAV, it will improve mosquito monitoring and control operations, which are complex and difficult to handle in urban areas.

Urbanization tends to result in the emergence of new vector-borne diseases and further exacerbation of other diseases [27–30], especially viral diseases transmitted by *Aedes* mosquitoes. In Taiwan, the most important vector organisms that infect the population with dengue fever and are closely connected to urbanization are *Ae*. *aegypti* and *Ae*. *albopictus*. Both species attach to human beings in urban development. Their expansion is not only due to environmental factors (environmental change and warming), but more significantly linked to artificial structures such as urban drainage systems (sewage and rainwater). This is particularly the case when the beds of the ditches are uneven, preventing normal drainage or overflow of the corresponding drainage system. Puddles of stagnant water may form in these places, providing ready habitat for *Aedes* mosquitoes. These ditches have become the main breeding source for the spread of dengue fever.

Our inspection using UGVs found that the ditches in Kaohsiung city were a breeding source for vector mosquitoes. Because Kaohsiung is a metropolitan city with a mix of old and new building developments, and the previous drainage system was rarely modified when old neighborhoods were rebuilt, the ditches in these areas were prone to ponding and siltation. Years later, the water in the recesses of these ditches turned them into large breeding sources for *Aedes* mosquitoes. Our findings are similar to previous studies showing in the urbanization processes create suitable habitats for *Aedes* mosquitoes [13,14,31], but there are few studies that have systematically investigated the risk factors to dengue epidemics posed by urbanization. This will be an important research topic in the future. Furthermore, the optimization of dengue control relies on sufficient information about the ecology of *Aedes* mosquitoes. The vector mosquito density map or positive ditch map obtained after inspection by UGVs shows the geographic distribution of risk factors for dengue outbreaks, thus providing a visual representation for epidemic prevention units to quickly summarize disease cluster information. Urbanization-related epidemic prevention measures for dengue transmission derived from such data should be applicable to many tropical countries with less access to investigation technology.

In urban areas with vector-borne diseases and residual transmission, effective vector control measures are necessary to reduce pathogen transmission. Dengue control can be achieved through identifying, monitoring, and treating aquatic habitats that support the development of vector mosquitoes. Recent developments in UGVs allow for accurate surveillance of mosquito habitats and implementation of interventions. The field is rapidly evolving, and new technology is addressing urbanization concerns. While technology continues to evolve in this field, UGVs are most effective when used in conjunction with other methods such as field surveys and control measures. The use of UGVs has the potential to enhance and target existing activities, providing more tools for mosquito surveillance and control.

The five administrative districts included in this study represent dengue hot spots in Kaohsiung city. Because estimated GI values differed between districts, we investigated likely causes and tried to determine which districts would be in greater need of attention in terms of mosquito control. We found that the districts of Nanzi, Fengshan, and Qianzhen had a GI that was higher than the average (GI_avg_ = 0.52), indicating a higher mosquito density in the field (Fig 5). This may be attributed to more positive ditches in these administrative districts (Fig 4A), which appear to be more conducive to mosquito breeding. Lingya had only one positive ditch and the lowest GI. After spraying insecticides and using other preventative epidemic control methods, the GI of each district was significantly reduced, with the exception of Fengshan which still had a relatively high GI. This may be because the *Aedes* mosquitoes in this area are resistant to insecticides [32], in which case other effective anti-epidemic actions may need to be employed to reduce the density of vector mosquitoes, e.g., release of *Wolbachia*-infected mosquitoes [33]. Taken together, the presence of favorable habitats for vector spreaders in positive ditches highlights the need for government attention and management to prevent these locations from becoming a source of dengue outbreaks.

Intelligent unmanned vehicles are gaining popularity and becoming increasingly researched by people. Despite this, certain potential shortcoming and limitations may impact their reliability in complex working environments and widespread use. For instance, factors such as battery life and control transmission wire length can affect the UGV’s detection time and range. To address these limitations, efforts could be made to reduce the UGV’s weight for ease of operation and enhance its capacity to support multiple sensors with varying resolutions to improve video quality. Additionally, operating the UGV requires professional knowledge, but future advancements in automated inspection paths will overcome this challenge. To further increase the reliability of UGV usage, advancements in sensors, autonomy, mobility, and AI are necessary. Overall, people are enthusiastic about the development of unmanned devices for vector mosquito control and their expectations continue to grow.

## Conclusion

Kaohsiung is a rapidly urbanizing and international city. Changes in urban infrastructure, such as the planning or construction of sewers, has resulted in the siltation of ditches. We used UGVs for live observation of dengue vector mosquitoes live in these habitats and found larval to adult stages of mosquitoes in a large percentage of ditches, indicating that Kaohsiung’s sewer systems constitute a hidden breeding source that cannot be ignored and must be controlled at all times. Failure to do so promotes the formation of dengue fever outbreak sources. In the future, new technologies (unmanned vehicle system) for the prevention of dengue fever could be widely used to raise epidemic prevention warning signals as soon as possible to effectively reduce the spread of dengue fever.

## Supporting information

S1 Table UGV specifications. (DOCX)

S1 Fig Analysis of the proportion of dengue vector positive containers in Kaohsiung city. The data is a cumulative average from January to October 2018. Source: Kaohsiung city Government website [19].
(TIF)

S1 Video Image of *Aedes* larvae in a ditch. (MP4)

S2 Video Image of *Aedes* adult mosquito and pupae in a ditch. (MP4)
