# Supplementary material for: Use of unmanned ground vehicle systems in urbanized zones: A study of vector Mosquito surveillance in Kaohsiung
Source: PLoS Negl Trop Dis. 2023 Jun 8;17(6):e0011346. doi: 10.1371/journal.pntd.0011346 (PMC10249801; doi:10.1371/journal.pntd.0011346)
Supplement: S1 Table — (DOCX) [file pntd.0011346.s001.docx]

**S1** **Table. UGV specifications.**

| **Crawling robot** | | **Wire-controlled cable car** | |
| --- | --- | --- | --- |
| **Rotation ability** | 360° | **Total length of control wire** | 200 m |
| **Working temperature** | -20 to 55℃ | **Number of wheels** | 4 wheels |
| **Waterproof ability** | IP68 | **Number of batteries** | 1 (exchangeable) |
| **Number of wheels**  **(Wheel diameter)** | 4 or 6 wheels  (13 or 23 cm) | **Control line take-up** | Automatic take-up |
| **Operation time** | 8 hours  (single battery) |  | |
| **Crawling slope** | Max 30° | **Real-time monitoring system** | |
| **Image sensor** | ¼ Sony CCD | **Software system** | Windows |
| **Active light source** | 4 high LED light | **Appearance type** | Integrated box type carry case |
| **Resolution** | 625 TVL | **Control axis** | 2 groups (lens and device orientation) |
| **Autofocus function** | 120x  (10x optical zoom, 12x digital zoom) | **Data storage device** | 64 GB hard drive |
