# Supplementary figures and images for: Use of unmanned ground vehicle systems in urbanized zones: A study of vector Mosquito surveillance in Kaohsiung
Source: PLoS Negl Trop Dis. 2023 Jun 8;17(6):e0011346. doi: 10.1371/journal.pntd.0011346 (PMC10249801; doi:10.1371/journal.pntd.0011346)

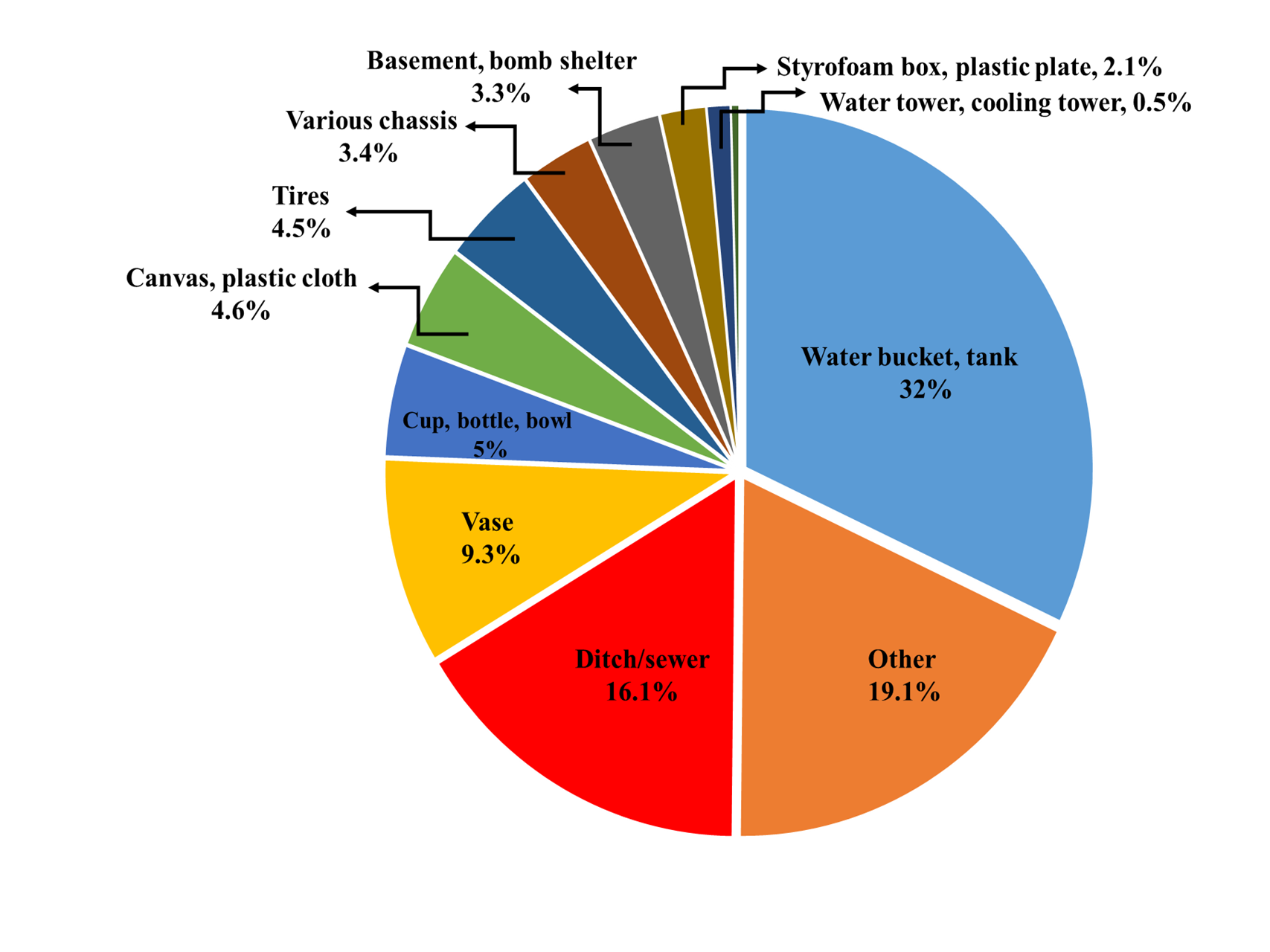

Supplement: S1 Fig — The data is a cumulative average from January to October 2018. Source: Kaohsiung city Government website [19]. (TIF) [file pntd.0011346.s002.tif]
